# Indents

**Published:** 1903-02-15

**Authors:** 


### INDENTS.

☞ **Brief Articles of Technical or Scientific Value will receive notice and comment. Contributions invited.**

**Fever Blisters.** Paint the blister with a solution of twelve grains of salicylic acid in one ounce of flexible Collodion.—O. L. Peak in *Alkaloidal Clinic.*

**To Improve Rubber Cups for Polishing Teeth.** To prevent the accumulation of air and the consequent difficulty of filling the rubber cups with pumice, punch a small hole in the side of the cup with the rubber dam, punch as near as practicable to the shoulder of the mandrel. Use the largest hole in the Ainsworth rubber dam punch.—A. C. Cameron, *Cosmos.*

**Sensitive Buccal and Labial Cavities.** Adjust the rubber dam and retain it in position with a suitable clamp. Dry the cavity with absorbent cotton and flood it with Justi's cement liquid, allowing the liquid to remain for twenty or thirty seconds. It is sometimes necessary to repeat the application using a small pledget of cotton dipped in the liquid, wiping out the cavity thoroughly and flood again, allowing the liquid to remain while the cavity is being prepared in the usual way.—*Review.*

**To Remove Posts Cemented into Roots.** Apply the rubber dam to isolate the tooth and protect the patient from inhaling the chemical fumes. Saturate a pledget of cotton with ammonia water and apply to the cement. As the cement softens remove it with a small sharp-pointed instrument. Continue application and instrumentation until sufficient of the post is exposed so that it may be grasped with a pair of pliers, when it can be removed by careful manipulation.—T. L. Pepperling, in *Dental Era*.

**Electric Ozonation.** Dr. G. Lenox Curtis has a paper in the August *Digest*, describing a method of curing neuralgia and other nerve pains by passing a current of electricity through the body by means of a "Geisler Vacuum Tube." His theory is that the current as it passes through the tissues generates or carries ozone into the diseased structures, or into the blood, and that it acts germicidally to destroy the pathogenic influences and to stimulate the nutrition in tissues abnormally depressed. He cites a number of remarkable cures in evidence; but the explanation of the method of treatment or the reasons for it are not very lucid. If it will accomplish what is claimed for it by the author it is certainly a brand new discovery of the greatest value.

**Setting a Banded Crown Painlessly.** When the parts are dried take a small probe and dip it in carbolic acid ninety-five per cent. then pass the end of the probe under the free margin of the gum, and carry it around the root so that the acid comes in contact with the surface of the gum touched by the band of the crown. This will not only anesthetize the gum and peridental membrane sufficiently to permit the band to go on painlessly, but it will sear the gum over so as to prevent to a large degree the seeping of moisture between the gum and the root. After setting the crown bathe the gum surrounding it with alcohol, to neutralize any permanent effect of the carbolic acid—particularly if too much has been used or it has touched the gum some distance away from the crown—*Review*.

